# Recommendations for management of infants and young children with achondroplasia: Does clinical practice align?

**DOI:** 10.1186/s13023-025-03621-7

**Published:** 2025-03-11

**Authors:** Encarna Guillen-Navarro, Moeenaldeen AlSayed, Inês Alves, Tawfeg Ben-Omran, Silvio Boero, Valérie Cormier-Daire, Brigitte Fauroux, Svein Fredwall, Melita Irving, Philip Kunkel, Christian Lampe, Ekkehart Lausch, Mohamad Maghnie, Klaus Mohnike, Geert Mortier, Zagorka Pejin, Marco Sessa, Sérgio B. Sousa

**Affiliations:** 1 Department of Pediatrics and Medical Genetics Section, Virgen de la Arrixaca University Hospital, IMIB-Pascual Parrilla, University of Murcia, Murcia, Spain; 2 CIBERER-ISCIII, Madrid, Spain; 3 Department of Medical Genomics, Center for Genomic Medicine, King Faisal Specialist Hospital and Research Centre, Riyadh, Kingdom of Saudi Arabia; 4 Faculty of Medicine, Alfaisal University, Riyadh, Kingdom of Saudi Arabia; 5 Comprehensive Health Research Centre, School of Health and Human Development, University of Évora, Évora, Portugal; 6 Division of Genetics and Genomic Medicine, Sidra Medicine and Hamad Medical Corporation, Doha, Qatar; 7 Istituto Giannina Gaslini, Genoa, Italy; 8 Hôpital Necker, Paris, France; 9 Pediatric Non-Invasive Ventilation and Sleep Unit, AP-HP, Necker Enfants Malades University Hospital and EA7330 VIFASOM Paris University, Paris, France; 10 TRS National Resource Centre for Rare Disorders, Sunnaas Rehabilitation Hospital, Nesodden, Norway; 11 Guy’s and St Thomas’ NHS Foundation Trust, London, UK; 12 Department of Neurological Surgery, University Medical Center Hamburg-Eppendorf, Hamburg, Germany; 13 Clinic of Child and Youth Medicine, University Hospital Mannheim, Mannheim, Germany; 14 Section of Pediatric Genetics, Medical Center, University of Freiburg, Freiberg, Germany; 15 Department of Pediatrics, IRCCS Istituto Giannina Gaslini, Genoa, Italy; 16 Department of Neuroscience, Rehabilitation, Ophthalmology, Genetics, Maternal and Child Health, University of Genova, 16147 Genoa, Italy; 17 Children’s Hospital, Otto-von-Guericke-University Magdeburg, Magdeburg, Germany; 18 Center for Human Genetics, University Hospital Leuven and KU Leuven, Leuven, Belgium; 19 Patient Advocate, AISAC, Milano, Italy; 20 Medical Genetics Unit, Hospital Pediátrico, Centro Hospitalar e Universitário de Coimbra, Coimbra, Portugal; 21 Faculty of Medicine, University Clinic of Genetics, Universidade de Coimbra, Coimbra, Portugal

**Keywords:** Achondroplasia, European Achondroplasia Forum, Infants, Young children, Guiding principles, Management, Recommendations

## Abstract

**Background:**

Achondroplasia is one of the most prevalent forms of skeletal dysplasia. Lifelong follow-up by an experienced multidisciplinary team is required, particularly during the first 2 years. In 2021, international consensus recommendations and guiding principles were published by two groups.

**Methods:**

We undertook two exploratory surveys to investigate awareness of the recommendations for management of children with achondroplasia among healthcare professionals (HCPs) and parents. We also assessed how well clinical practice aligns with the recommendations.

**Results:**

Awareness of guidance was high among HCP respondents but low among parent respondents. Clinical practice largely aligned with international guidance; however, there was not complete alignment with all recommendations with several rating “somewhat” or “not at all aligned”. For infants, these included referral to skeletal dysplasia centre or an HCP with expertise in achondroplasia after diagnosis, provision to parents of early information on positioning and handling, mandatory evaluation for cervicomedullary compression at each medical evaluation, sleep study within the first year of life, and adherence to national immunisation programmes. For children aged 2–5 years, these included annual audiology assessment, encouraging parents to keep children active and learn early healthier nutritional habits, consultation with a paediatric orthopaedic spine specialist if a kyphosis has not resolved within a year, consultation with a paediatric orthopaedic surgeon in the case of progressive genu varum, discussion of limb lengthening procedures, and regular dental assessments.

**Conclusions:**

Further research is needed to understand the reasons for deviation from recommendations. Efforts to increase alignment with recommendations could include disseminating to the wider group of specialties that care for people with achondroplasia and seeking alternative approaches to current organisation of care, such as hub-and-spoke models. Raising awareness of the guidance among parents could be achieved by adapting materials for a non-HCP audience, translation and sharing through patient advocacy groups.

**Supplementary Information:**

The online version contains supplementary material available at 10.1186/s13023-025-03621-7.

## Background

Achondroplasia is one of the most prevalent forms of skeletal dysplasia, occurring with a frequency of 1 in 10,000–30,000 population [1–3]. The majority of patients with achondroplasia have one of two variants in the fibroblast growth factor receptor 3 (*FGFR3*) gene, resulting in the amino acid change p.Gly380Arg [1–3]; in 75–80% of cases the mutation occurs de novo [1–3]. Achondroplasia is characterised by disproportionate short stature, genu varum, exaggerated lumbar lordosis, narrowing of the lumbar interpedicular distance, macrocephaly, midface hypoplasia, frontal bossing, and reduced size of the foramen magnum [1–3]. Complications may include pain, cervicomedullary compression in the first few months of life due to reduced size of the foramen magnum, obstructive sleep apnoea due to midface hypoplasia, and recurrent otitis media, which can result in conductive hearing loss [1–3]. Achondroplasia is usually diagnosed at birth [1] or in early infancy, although prenatal recognition has become more frequent and more accurate [3–6]. Diagnosis should not be delayed, as some complications can be prevented through assessment in early infancy [4, 7].

### Management of achondroplasia

Management of achondroplasia is lifelong and requires an experienced multidisciplinary team (MDT), with close monitoring during the first 2 years in particular [3, 8, 9], and continued monitoring throughout life. The primary goals are to anticipate, identify and treat associated co-morbidities and provide education and support to encourage a future healthy lifestyle, positive self‑esteem and mental health, autonomy, and independence [8]. Complications are treated through symptomatic management, surgical intervention, and lifelong follow-up care [1].

### Current guidance

In 2021, the European Achondroplasia Forum (EAF) published the first European consensus on guiding principles of management for achondroplasia [8]. The EAF is an independent network of specialists managing achondroplasia, which aims to improve overall care through collaboration and sharing of best practice. The Steering Committee includes representation from across Europe and the Middle East and comprises clinical geneticists, paediatric endocrinologists, orthopaedic surgeons, a general practitioner specialised in the care of adults with achondroplasia, a neuropaediatrician, a neurosurgeon, a patient advocacy group (PAG) representative and a sleep specialist. The EAF guiding principles for the management of achondroplasia are shown in Table 1.

**Table 1:** European Achondroplasia Forum guiding principles of management for achondroplasia [8]

A	Achondroplasia is a lifelong condition requiring lifelong management by an experienced MDT, led by physicians/clinicians experienced in achondroplasia management. Close monitoring during the first 2 years of life is critical
B	When a diagnosis of achondroplasia is made or suspected, either in utero or after birth, the family should be referred as soon as possible to a physician experienced in achondroplasia to discuss the prognosis and management of the condition
C	Decisions around management should be made in the MDT setting jointly with the person with achondroplasia and/or their family
D	The primary goals of management are to enable anticipation, identification and treatment of problems, provide education and support to encourage a healthy lifestyle, positive self‑esteem and mental health, autonomy and independence
E	Patients should have access to a variety of adaptive measures, support to ensure proper usage and access to approved treatment options as they become available
F	Regular monitoring in adolescence and adulthood should continue under an MDT with expertise in management of achondroplasia. Care should include genetic counselling, transition to adulthood, psychosexual wellbeing and management of pregnancy

Also in 2021, 55 international experts from 16 countries and five continents published a consensus statement and recommendations for management of achondroplasia [9]. This statement aimed to improve and standardise care for children and adults with achondroplasia worldwide to optimise clinical outcomes and quality of life. It includes recommendations specific to management of achondroplasia for infants and for young children [9], and has been translated into 14 languages [10].

The EAF sought to understand how well the guiding principles [8] and international consensus recommendations [9] have been disseminated among healthcare professionals (HCPs) and parents of children with achondroplasia, and how well clinical practice aligns with the international recommendations.

## Methods

Two exploratory surveys were developed, one designed to gain the perspective of HCPs and the other the perspective of parents of children with achondroplasia. The aim of the surveys was to assess awareness of and alignment with the recommendations for management of achondroplasia in infants and young children in clinical practice.

To ensure rigour in the development of the survey, a specialist in survey design and methodology was consulted, the survey was pilot tested on four people (ENG, KM, IA, MS) and updated based on feedback prior to wider circulation. The parent survey was translated into selected languages including French, German, Italian, Spanish and Portuguese to reduce language bias. All translated surveys were checked by native speakers familiar with the subject matter. Detailed definitions of wording were not provided in the survey, so a level of personal interpretation was permitted. The exploratory surveys were designed to capture a snapshot of current practices in responding centres and the lived experience of children with achondroplasia according to their parents/caregivers; as such, the surveys were not validated.

The HCP survey was distributed via email to the EAF Steering Committee, and the parent survey was distributed via email to a list of contacts from patient advocacy groups across Europe, provided by the PAG representative who supported the development of the survey (IA). Both surveys were also made available on the EAF website (www.achondroplasiaforum.com). All recipients were asked to circulate the survey to their colleagues and personal networks. We therefore cannot quantify how many people received the survey and not all who answered the survey necessarily completed it. The distribution of the surveys was not targeted and was intended to gather the opinions of people who had expressed in interest in the EAF.

The results of the exploratory surveys were collated and presented at an online open workshop held in October 2022. The workshop was advertised through the EAF website and mailing list, as well as through the EAF Steering Committee’s personal contacts, professional networks and by PAGs. The workshop was open for all to attend and included participation from 45 HCPs and six PAG representatives from a total of 15 countries. The participants in the session discussed the results of the surveys, assessed challenges and proposed strategies to improve the management of infants and young children with achondroplasia.

## Results

### Respondents

**Fig. 1:**
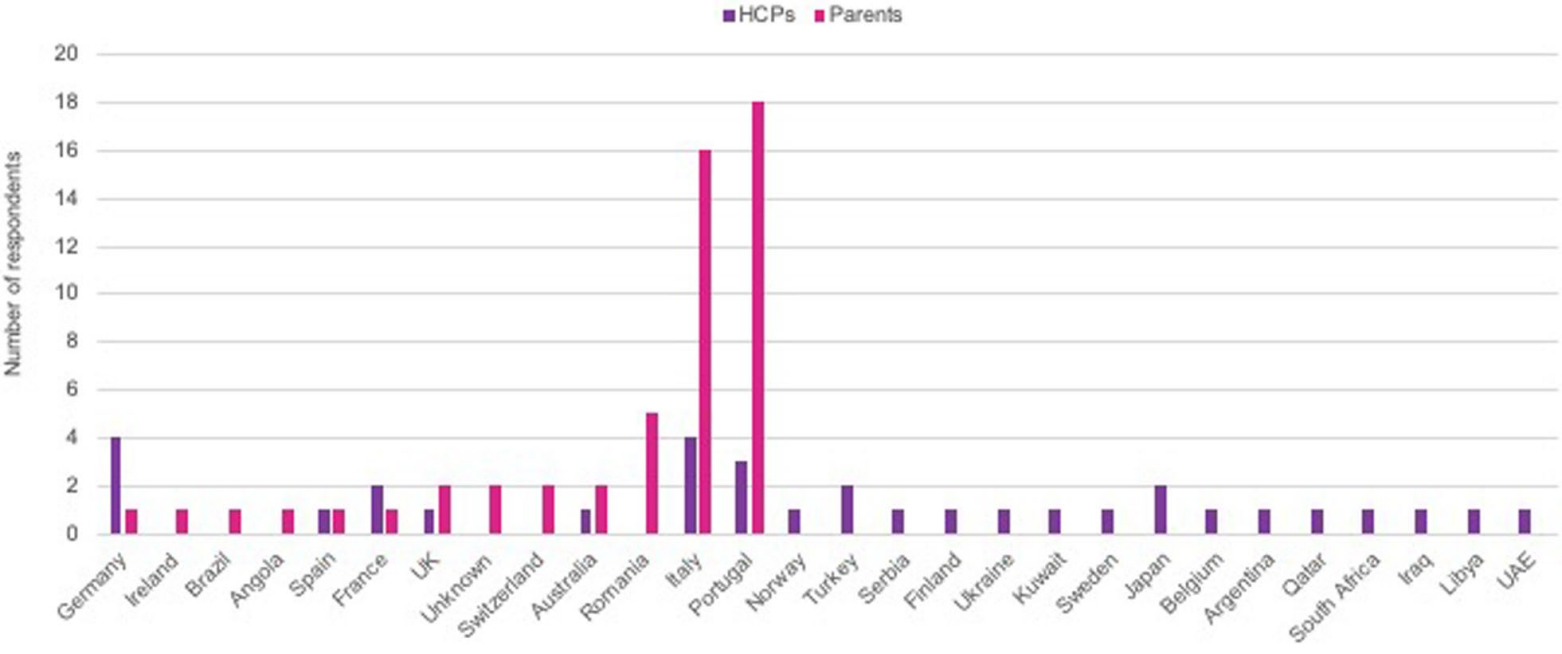
Respondents by country

**Fig. 2:**
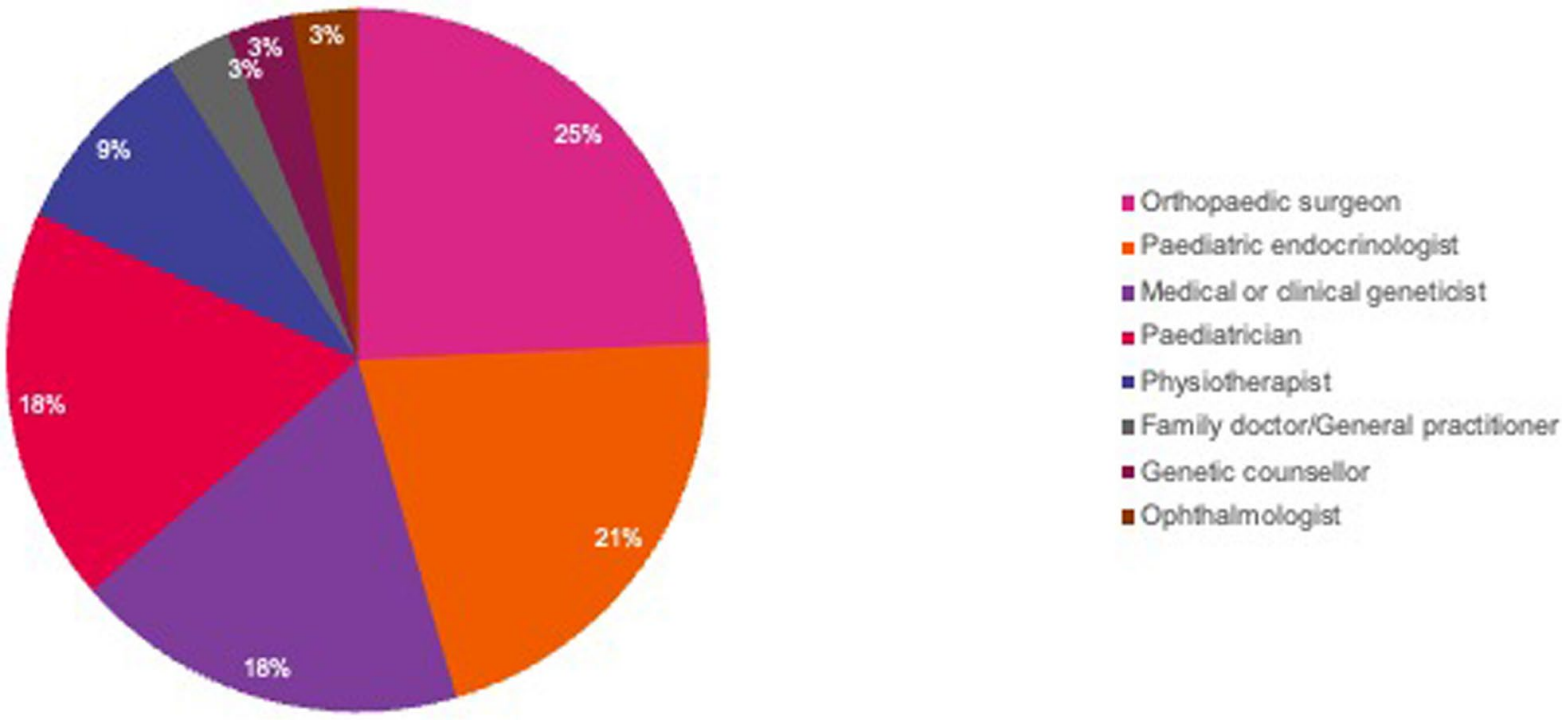
Healthcare professional respondents by speciality

A total of 33 HCPs from 22 countries and 53 parents of children with achondroplasia from 13 countries responded (Fig. 1). The responding HCPs were from a variety of specialties, mainly orthopaedic surgeons (n = 8, 25%), paediatric endocrinologists (n = 7, 21%), paediatricians (n = 6, 18%), medical or clinical geneticists (n = 6, 18%), but also physiotherapists (n = 3, 9%), and one each (3%) of family doctor/general practitioner (GP), ophthalmologist, genetic counsellor and paediatrician (Fig. 2).

Most responding parents were of average stature (n = 45, 85%); six (11%) had achondroplasia and one (2%) was of average stature with a partner with achondroplasia. One respondent did not answer this question.

**Fig. 3:**
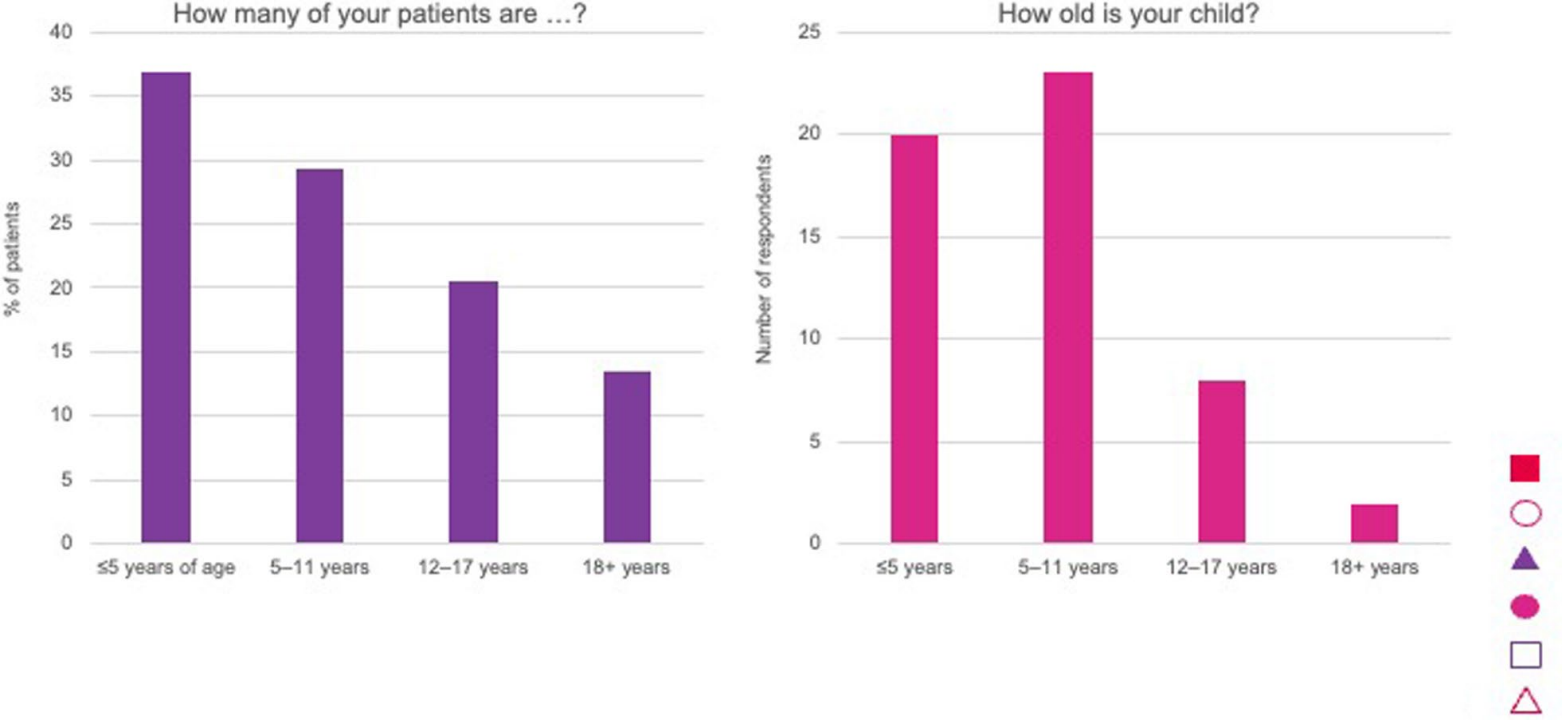
Age of patients managed by HCP responders, and age of children of parent responders

Most HCPs (n = 25) were based in academic institutions, specialist achondroplasia centres or university hospitals, with the remainder from general hospitals (n = 6); two were based in both academic institutions and general hospital. None worked in primary care (the responding GP is based at a National Resource Centre). Similarly, most parents (n = 35) reported that their children were managed in academic institutions, specialist achondroplasia centres or university hospitals followed by general hospitals (n = 16), with one managed in primary care. One respondent specified none, as the child was older than 18 years. The children managed by HCPs were < 5 years (37%), 5–11 years (29%), 12–17 years (20.5%) and ≥ 18 years (13.5%) (Fig. 3). More children of responding parents were aged 5–11 years old (n = 23, 43%) than < 5 years (n = 20, 38%); 12–17 years (n = 8, 15%) or ≥ 18 years (n = 2, 4%) (Fig. 3).

The following analysis of the survey results applies to the respondents only and are not intended to reflect the experience of the whole achondroplasia community.

### Awareness of international consensus recommendations and EAF guiding principles

**Fig. 4:**
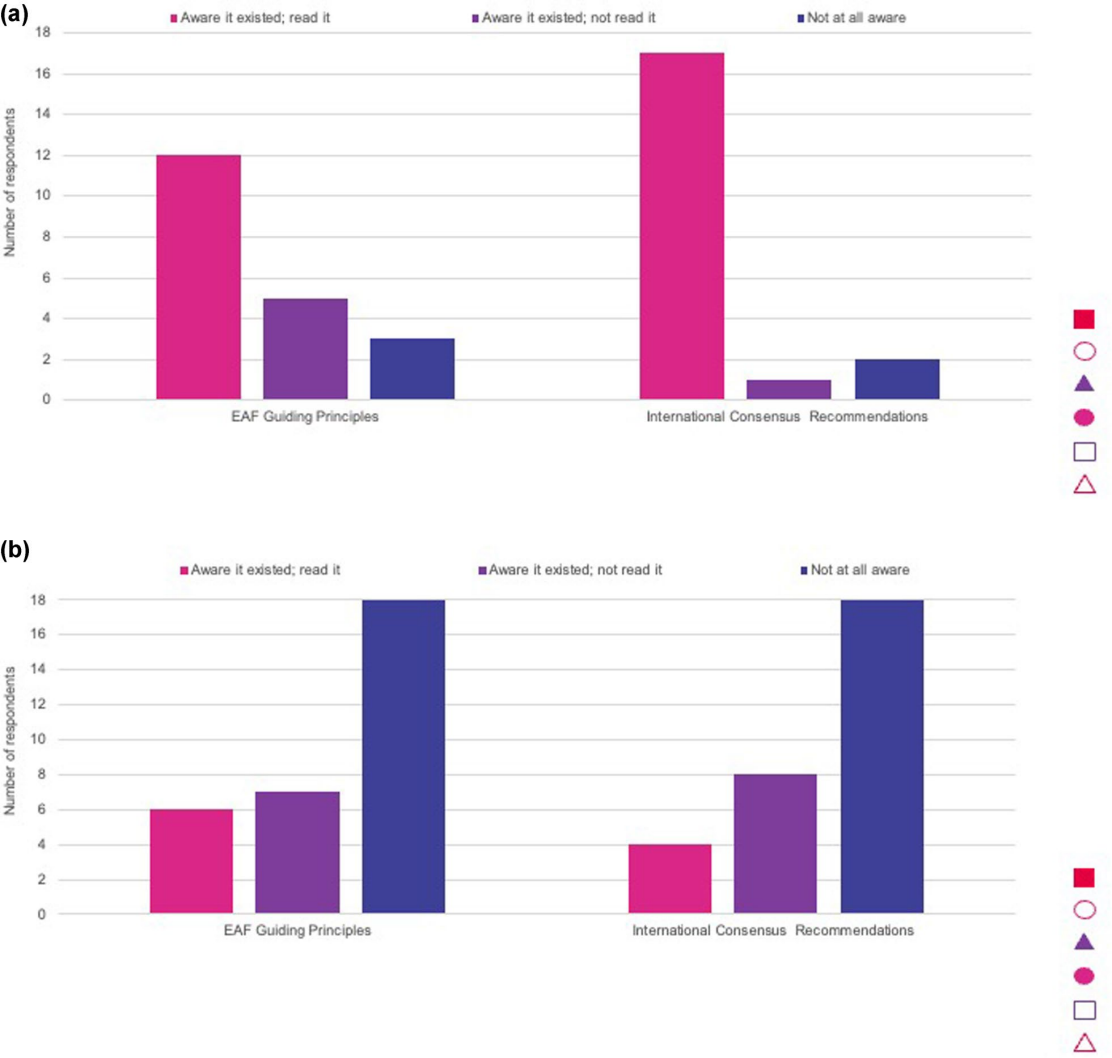
Awareness of International Consensus Recommendations and European Achondroplasia Forum (EAF) Guiding Principles for Achondroplasia among, **a** healthcare professionals and **b** parents of children with achondroplasia

The survey results showed that most HCPs who responded had read the international consensus recommendations and were generally aware of the EAF guiding principles (Fig. 4). An ophthalmologist noted that he/she had no knowledge of the guidance. Parent respondents were typically not aware of the guiding principles or recommendations (Fig. 4), but they did feel they would be helpful to them in understanding their child’s care.

### Alignment with international consensus recommendations in clinical practice

The survey for HCPs found that clinical practice among respondents is largely aligned with international consensus guidance for managing achondroplasia in infants and young children; however, some lack of alignment was identified (Table 2). For infants, notable recommendations scoring “somewhat” or “not at all aligned” included referral to skeletal dysplasia centre or an HCP with expertise in achondroplasia after diagnosis, provision to parents of early information on positioning and handling, mandatory evaluation for cervicomedullary compression at each medical evaluation, polysomnography or respiratory polygraphy within the first year of life, and adherence to national immunisation programmes.

**Table 2:** Healthcare Professionals: Alignment with international consensus recommendations [9] for management of achondroplasia in infants (< 2 years) and young children (2–5 years)

Recommendation	Alignment in clinical practice		
	Completely n (%)	Somewhat n (%)	Not at all n (%)
**Infancy (n = 20 respondents)**			
24. Infants with ACH should be referred to a skeletal dysplasia reference centre or an HCP with expertise in ACH as soon as the diagnosis is made	14 (70)	6 (30)	0
25. All children with ACH should receive regular follow-up by a MDT, guided by a HCP professional with expertise in ACH. Close monitoring in the first 2 years of life is important	16 (80)	3 (15)	1 (5)
26. Parents of infants with ACH should be provided with specific charts and a growth parameters register (height, weight and head circumference) for management follow-up	16 (80)	4 (20)	0
27. Gross and fine motor developmental milestones are different in infants with ACH as compared with average stature, age-matched peers. Infants with ACH should be assessed for the development of gross, fine motor and early communication skills using ACH-specific screening tools. If developmental delay is observed, MRI of the head and spine and an assessment by a paediatrician and/or neurologist should be considered	15 (75)	4 (20)	1 (5)
28. Parents should be provided with early information on positioning and handling infants with ACH, including avoidance of early sitting and appropriate options for car seats and prams	12 (60)	8 (40)	0
29. Careful evaluation for cervicomedullary compression is mandatory at each medical evaluation in infants and young children with ACH. Signs and symptoms of cervicomedullary compression include motor regression or delayed milestone acquisition, apnoea, difficulty swallowing, poor weight gain, clonus, abnormal reflexes and weakness. Concerning signs or symptoms should be evaluated urgently by a paediatric neurosurgeon	14 (70)	6 (30)	0
30. An increased incidence of sleep-disordered breathing is present in infants with ACH and parents should be informed about typical signs of sleep apnoea. A polysomnography study should be performed when respiratory problems are obvious or suspected but, in any case, completed during the first year of life for all infants with ACH	12 (60)	8 (40)	0
31. Hearing evaluation is recommended in infants with ACH at an early stage and should be monitored longitudinally	14 (70)	5 (25)	1 (5)
32. Recurrent and chronic otitis media are common in infants with ACH and early referral to an otolaryngologist (ENT specialist) should be considered	15 (75)	5 (25)	0
33. Infants with ACH should receive regular vaccinations according to national immunisation programmes	16 (80)	2 (10)	2 (10)
**Early childhood (n = 17 respondents)**			
53. Middle ear effusions are common in children with ACH and can impair hearing. This condition should be screened for with audiology assessments at least annually in early childhood and, if there are concerns, the children should be referred to ENT for consideration of grommets	10 (58)	6 (35)	1 (5.9)
54. If there is speech and language delay in the ACH development milestones, then the child should be referred for speech and language therapy	12 (70.6)	4 (23.5)	1 (5.9)
55. OSA can be a common complication of ACH that presents with apnoea or snoring. Parents should be informed of these symptoms and clinicians should ask about OSA during consultations	14 (82.4)	3 (17.6)	0
56. A healthy lifestyle with emphasis on physical activity and healthy eating should be encouraged during each consultation of a child with ACH	12 (70.6)	5 (29.4)	0
57. Infants and children with ACH who are noted to be developing behind their peers with ACH when assessed using condition-specific milestone achievement recommendations should be referred to physiotherapists, occupational therapists and speech pathologists with skills in this area	12 (70.6)	5 (29.4)	0
58. Children with ACH should be reviewed by a physiotherapist and/or occupational therapist with skills in this area to support the development of independence skills, particularly in the area of self-care activities	11 (64.7)	5 (29.4)	1 (5.9)
59. Trips and falls might be common when the child starts to walk. Parents should be encouraged to keep the child active but be aware that they might trip and fall more frequently than children of average stature so appropriate precautions should be taken	8 (47.1)	8 (47.1)	1 (5.9)
60. Careful monitoring of the spine should be undertaken in children with ACH. If a kyphosis has not resolved within a year or is progressive in a child who is walking, consultation with a paediatric orthopaedic spine surgeon with experience in ACH is recommended	10 (58.8)	7 (41.2)	0
61. Genu varum might start to develop in this age group (age 2–12 years) and become pronounced. If progressive, evaluation by a paediatric orthopaedic surgeon with experience in ACH should be considered	10 (58.8)	7 (41.2)	0
62. The possibility of children with ACH undergoing limb lengthening procedures might be discussed and explained to the patient, family and caregivers at this stage. Psychological consultation is advised before undertaking limb lengthening procedures	7 (41.2)	5 (29.4)	5 (29.4)
63. During every consultation, the medical team should actively investigate for the presence of pain and/or fatigue in children with ACH. If these symptoms are present, a clinical assessment to determine the cause should be conducted	12 (70.6)	4 (23.5)	1 (5.9)
64. Surgical interventions should only be performed by surgeons with expertise in ACH and decisions should be made in conjunction with the input of the full ACH MDT	11 (64.7)	5 (29.4)	1 (5.9)
65. Regular dental assessments for all children with ACH should be encouraged and referral to orthodontics should be made when needed	7 (41.2)	9 (52.9)	1 (5.9)
66. Children with ACH should use well-fitted car seats for as long as possible and according to local safety standards	12 (70.6)	4 (23.5)	1 (5.9)

Percentages may not add up to 100 due to rounding

For children aged 2–5 years, notable recommendations scoring “somewhat” or “not at all aligned” included annual audiology assessment, encouraging parents to keep children active but aware that they may trip and fall more frequently as they start to walk, consultation with a paediatric orthopaedic spine specialist if a kyphosis has not resolved within a year, consultation with a paediatric orthopaedic surgeon in the case of progressive genu varum, discussion of limb lengthening procedures, and regular dental assessments.

## Discussion

Our survey found that most HCPs who responded to the exploratory survey had read the international consensus recommendations and were generally aware of the EAF guiding principles, while parent respondents were typically not. Management of achondroplasia in infants and young children was largely aligned with international consensus guidance, but some variation in alignment was identified.

### Awareness of recommendations and guiding principles

High awareness of the recommendations and guiding principles among HCP respondents is unsurprising, as most were based in academic institutions, specialist achondroplasia centres or university hospitals. However, as management and follow up may be shared between expert centres and local healthcare providers, the latter also need to be aware of the recommendations. Efforts therefore need to be made to disseminate recommendations to the wider group of specialties in the care of people with achondroplasia. This could be achieved through presenting the recommendations at international conferences and patient organisation meetings, participation in discussions about guidance on best practice management, or dissemination through rare disease organisations and websites. Low awareness among parent responders is likely because the recommendations were developed for HCPs and have not been adapted or made available for parents of children with achondroplasia. It is also feasible that low awareness among parent responders could be aligned to limited referral to a skeletal dysplasia centre, or an HCP experienced in achondroplasia after diagnosis. Although the recommendations have been translated into 14 languages, this may not be enough, as one parent respondent noted that they had taken the action themselves to translate the recommendations to share within their network. Translating into further languages will be helpful to reach a broader parent audience and, with the collaboration of local patient organisations, developing summaries of the guiding principles and recommendations using terminology suitable for parents and other non-clinical stakeholders may be helpful to increase parent awareness.

### Alignment with international consensus recommendations

Varied alignment with the published recommendations for achondroplasia in practice may be caused by a range of factors including availability of expertise, structure of care, and organisational issues. Lack of complete alignment with recommendations around MDTs, monitoring and surgical interventions may reflect that specialists with an interest in achondroplasia are not locally available. This can lead to delays in referral. In some cases, local hospitals may not support HCPs to follow the recommendations and care may be devolved to local services, which are not always consistent or well versed in delivery of aspects of care such as evaluation of cervicomedullary compression, sleep studies and achondroplasia-specific orthopaedic surgery.

Guidance often focuses on management of complications of achondroplasia at expert centres, but the reference centre model may not be feasible for many settings and national centres are not officially endorsed in some countries. Some national centres are based on one speciality—for example, orthopaedics—where on-site access to other specialties may not be available. As is the case with many rare diseases, patients are typically scattered across a country and it can be difficult for them to access specialist care without travelling long distances to expert centres [11]. Clinicians for other rare diseases, such as haemophilia, have adopted hub-and-spoke models, in which a specialist centre provides the core care, supported by centres that may be more local to patients [11, 12]. A hub-and-spoke model for achondroplasia may involve a ‘reference centre’ with a core MDT team as the ‘hub’, supported by ‘competence centres’ that include additional specialties as ‘spokes’. This model would allow expertise to be collated at specialist centres, with a specialist in another hospital still considered part of the MDT. The hub-and-spoke model could also be supported through telemedicine, allowing patients to access required expertise without having to travel long distances. It should be acknowledged, however, that a hub-and-spoke model requires a large amount of coordination and may not be feasible in large countries or where logistical management is particularly challenging.

Clinician respondents aware of the recommendations may not feel they are locally relevant or appropriate to a specific child. In the example of discussing limb lengthening, clinicians may feel that this intervention is not appropriate in early childhood, so they may not fully align with the recommendation. It was also observed that four HCPs (all from different countries) reported aligning “somewhat” or “not at all” with the recommendation *Infants with achondroplasia should receive regular vaccinations according to national immunization programmes*. It is concerning that 30% of respondents only “somewhat align” with the recommendation *Careful evaluation for cervicomedullary compression is mandatory at each medical evaluation in infants and young children with achondroplasia […] Concerning signs or symptoms should be evaluated urgently by a paediatric neurosurgeon* (Table 2, no. 29). In addition, 40% of respondents only “somewhat” align with *An increased incidence of sleep-disordered breathing is present in infants with achondroplasia and parents should be informed about typical signs of sleep apnoea. A polysomnography study should be performed when respiratory problems are obvious or suspected but, in any case, completed during the first year of life for all infants with achondroplasia* (Table 2, no. 30). These are key factors in the management of achondroplasia in early childhood; an understanding of why these recommendations on critical aspects do not see universal alignment would be beneficial to improving care.

Another factor potentially limiting alignment with guidance among respondents is the fact it is based on expert consensus rather than clinical evidence, which is currently lacking. For example, the absence of evidence to define a schedule for MRI monitoring means that timing of scans varies between centres; this may also be affected by local/regional healthcare systems and available services. The international guidance is based on the best available evidence and a rigorous modified Delphi process involving 55 experts. Clinical trials for emerging drug treatments are already providing useful information, but, moving forward, it will be critical to develop further evidence around other interventions, including surgery, outcomes, and monitoring, such as real-world evidence from clinical practice through registries and post-marketing surveillance, so that HCPs are able to develop recommendations that are evidence- as well as eminence-based.

### Limitations

Conclusions from the survey findings should be interpreted with caution due to potential limitations in terms of reliability highlighted by some anomalous results and sources of potential bias.

We cannot verify the number of people who received the survey to compare with the number of respondents. Most responses were from two countries (Italy and Portugal) for the parent survey, and from three countries (Italy, Portugal, Germany) for the HCP survey (Fig. 1). Consequently, the results may be reflective only of the clinical practice of the individuals, and not of the wider country, or of the experience outside the countries of the respondents. Most respondents to both surveys were either from, or received follow up in, academic institutions, specialist achondroplasia centres and university hospitals, so their responses may represent closer to optimal practice than in the wider clinical community. Representation of general HCPs not experienced in achondroplasia was limited. For the parent survey, the respondents may be those who are more likely to seek expert care in specialist centres or adhere to guidance aligned with clinical practice recommendations.

There was limited dissemination of the parent survey by patient advocacy groups outside of Italy and Portugal, which may have impacted the data.

## Conclusions

Based on the EAF exploratory surveys, following publication of the international consensus recommendations and EAF guiding principles on management of achondroplasia, it was found that awareness of the guidance was high among HCPs respondents but low among parents respondents. Management in clinical practice largely aligned with international consensus recommendations, although complete alignment with some recommendations was not observed. The authors acknowledge limitations in the dissemination of the surveys and the potential bias of the results given the respondents were from limited countries and were from or received care in expert centres. Further research is needed to understand deviation from recommendations, including consideration of real-world evidence and local/country specific realities, as well as strengthening the evidence upon which guidance is based. Efforts need to be made to raise awareness of the guidance among parents.

## Supplementary Information

Additional file 1: Clinical management challenges in infants and young children with achondroplasia—Healthcare professionals survey.

Additional file 2: Clinical management challenges in infants and young children with achondroplasia—Parent survey.

## Data Availability

Not applicable.

## Abbreviations

EAF: European Achondroplasia Forum
*FGFR3*: Fibroblast growth factor receptor 3
HCP: Healthcare professional
MDT: Multidisciplinary team
MRI: Magnetic resonance imaging
OSA: Obstructive sleep apnoea
PAG: Patient advocacy group
