# Supplementary material for: Recommendations for management of infants and young children with achondroplasia: Does clinical practice align?
Source: Orphanet J Rare Dis. 2025 Mar 11;20:114. doi: 10.1186/s13023-025-03621-7 (PMC11895228; doi:10.1186/s13023-025-03621-7)
Supplement: Supplementary file 1 — Additional file 1: Clinical management challenges in infants and young children with achondroplasia—Healthcare professionals survey. [file 13023_2025_3621_MOESM1_ESM.pdf]

EAF
European Achondroplasia Forum

Clinical management challenges in infants and young children with achondroplasia – HCP survey

Survey objectives

- Identify the key clinical challenges in the management of achondroplasia in infants and young children
- Identify the key outcomes of importance for clinicians and families of children with achondroplasia
- Assess the alignment of clinical practice to recommendations

Please answer questions based on practice in your centre. If you are aware of differences elsewhere in your country, please add a comment

Name (optional):

General

1. Please enter your specialty:
- ☐ Family doctor/General practitioner
- ☐ Genetic counsellor
- ☐ Medical or clinical geneticist
- ☐ Neonatologist
- ☐ Neuropaediatrician
- ☐ Neurosurgeon
- ☐ Obstetrician
- ☐ Occupational therapist
- ☐ Orthopaedic surgeon
- ☐ Paediatric endocrinologist
- ☐ Paediatrician
- ☐ Physiotherapist
- ☐ Radiographer/Imaging specialist
- ☐ Rheumatologist
- ☐ Other (please specify):

2. Please enter your country:

3. What best describes your centre?
- ☐ Academic institution/Specialist achondroplasia centre/University hospital
- ☐ General hospital
- ☐ Primary care
- ☐ Other, please specify:

4. How many of your patients are:
- ☐ ≤5 years of age
- ☐ 5–11 years
- ☐ 12–17 years
- ☐ 18+ years

Key challenges in managing infants and young children

5. Which THREE complications of achondroplasia cause you the greatest challenge in the management of infants/young children?

	In infants ≤2 years	In children 2–5 years
Sleep-disordered breathing		
Otitis media		
Middle ear effusion		
Foramen magnum stenosis		
Thoracolumbar kyphosis		
Spinal stenosis		
Kyphosis		
Genu varum		
Obesity/Weight management		
Health-related quality of life factors (e.g., independent living, adaptive measures)		
Other (please specify):		

Please provide comment on why these complications cause you the greatest challenge:

6. How well do you think each key complication is managed in your centre?

	Extremely well	Well	Satisfactorily	Poorly	Very poorly
Sleep-disordered breathing					
Otitis media					
Middle ear effusion					
Foramen magnum stenosis					
Thoracolumbar kyphosis					
Spinal stenosis					
Kyphosis					
Genu varum					
Obesity/Weight management					
Health-related quality of life factors (e.g., independent living, adaptive measures)					
Other (please specify):					

Please provide comment on your response:

Recommendations and clinical practice

7. Prior to taking this survey, how aware were you of the existence of the EAF Guiding Principles for Management of Achondroplasia and the International Consensus Recommendations for the Management of Achondroplasia?

	EAF Guiding Principles	International Consensus Recommendations
Aware it existed, and have read it<br>Aware it existed but have not read it<br>Not at all aware		

Please comment:

8. How well aligned is your clinical practice to the following recommendations on management of achondroplasia in infants from the International Consensus Recommendations for the Management of Achondroplasia?

	Completely aligned	Somewhat aligned	Not at all aligned
**Management of achondroplasia in infants**			
Recommendation 24. Infants with achondroplasia should be referred to a skeletal dysplasia reference centre or a health-care professional with expertise in achondroplasia as soon as the diagnosis is made			
Recommendation 25. All children with achondroplasia should receive regular follow-up by a multidisciplinary team, guided by a health-care professional with expertise in achondroplasia. Close monitoring in the first 2 years of life is important			
Recommendation 26. Parents of infants with achondroplasia should be provided with specific charts and a growth parameters register (height, weight and head circumference) for management follow-up			
Recommendation 27. Gross and fine motor developmental milestones are different in infants with achondroplasia as compared with average stature, age-matched peers. Infants with achondroplasia should be assessed for the development of gross, fine motor and early communication skills using			

achondroplasia-specific screening tools. If developmental delay is observed, MRI of the head and spine and an assessment by a paediatrician and/or neurologist should be considered			
Recommendation 28. Parents should be provided with early information on positioning and handling infants with achondroplasia, including avoidance of early sitting and appropriate options for car seats and prams			
Recommendation 29. Careful evaluation for cervicomedullary compression is mandatory at each medical evaluation in infants and young children with achondroplasia. Signs and symptoms of cervicomedullary compression include motor regression or delayed milestone acquisition, apnoea, difficulty swallowing, poor weight gain, clonus, abnormal reflexes and weakness. Concerning signs or symptoms should be evaluated urgently by a paediatric neurosurgeon			
Recommendation 30. An increased incidence of sleep-disordered breathing is present in infants with achondroplasia and parents should be informed about typical signs of sleep apnoea. A polysomnography study should be performed when respiratory problems are obvious or suspected but, in any case, completed during the first year of life for all infants with achondroplasia			
Recommendation 31. Hearing evaluation is recommended in infants with achondroplasia at an early stage and should be monitored longitudinally			
Recommendation 32. Recurrent and chronic otitis media are common in infants with achondroplasia and early referral to an otolaryngologist (ear, nose and throat; ENT) specialist should be considered			
Recommendation 33. Infants with achondroplasia should receive regular vaccinations according to national immunization programmes			

Please comment on alignment/lack of alignment:

9. How well aligned is your clinical practice to the following recommendations on management of achondroplasia in childhood from the International Consensus Recommendations for the Management of Achondroplasia?

**Management in childhood**			
Recommendation 53. Middle ear effusions are common in children with achondroplasia and can impair hearing. This condition should be screened for with audiology assessments at least annually in early childhood and, if there are concerns, the children should be referred to ENT for consideration of grommets			
Recommendation 54. If there is speech and language delay in the achondroplasia development milestones, then the child should be referred for speech and language therapy			
Recommendation 55. Obstructive sleep apnoea (OSA) can be a common complication of achondroplasia that presents with apnoea or snoring. Parents should be informed of these symptoms and clinicians should ask about OSA during consultations			
Recommendation 56. A healthy lifestyle with emphasis on physical activity and healthy eating should be encouraged during each consultation of a child with achondroplasia			
Recommendation 57. Infants and children with achondroplasia who are noted to be developing behind their peers with achondroplasia when assessed using condition-specific milestone achievement recommendations should be referred to physiotherapists, occupational therapists and speech pathologists with skills in this area			
Recommendation 58. Children with achondroplasia should be reviewed by a physiotherapist and/or occupational therapist with skills in this area to support the development of independence skills, particularly in the area of self-care activities			
Recommendation 59. Trips and falls might be common when the child starts to walk. Parents should be encouraged to keep the child active but be aware that they might trip			

and fall more frequently than children of average stature so appropriate precautions should be taken			
Recommendation 60. Careful monitoring of the spine should be undertaken in children with achondroplasia. If a kyphosis has not resolved within a year or is progressive in a child who is walking, consultation with a paediatric orthopaedic spine surgeon with experience in achondroplasia is recommended			
Recommendation 61. Genu varum might start to develop in this age group (age 2–12 years) and become pronounced. If progressive, evaluation by a paediatric orthopaedic surgeon with experience in achondroplasia should be considered			
Recommendation 62. The possibility of children with achondroplasia undergoing limb lengthening procedures might be discussed and explained to the patient, family and caregivers at this stage. Psychological consultation is advised before undertaking limb lengthening procedures			
Recommendation 63. During every consultation, the medical team should actively investigate for the presence of pain and/or fatigue in children with achondroplasia. If these symptoms are present, a clinical assessment to determine the cause should be conducted			
Recommendation 64. Surgical interventions should only be performed by surgeons with expertise in achondroplasia and decisions should be made in conjunction with the input of the full achondroplasia multidisciplinary team			
Recommendation 65. Regular dental assessments for all children with achondroplasia should be encouraged and referral to orthodontics should be made when needed			
Recommendation 66. Children with achondroplasia should use well-fitted car seats for as long as possible and according to local safety standards			

Please comment on alignment/lack of alignment:

10. Which three recommendations from the International Consensus Recommendations for the Management of Achondroplasia do you think cause the greatest challenge to follow in clinical practice, and why?

11. What plans do you have, if any, to update your practice to be more aligned with recommendations?

Outcomes in infants and young children

12. From your clinical perspective, what do you consider to be the most important outcomes in the management of achondroplasia in infancy and childhood? Please select up to THREE options

	In infants ≤2 years	In children 2–5 years
Decompression of the foramen magnum (if indicated)<br>Achievement of ACH-specific growth milestones<br>Achievement of ACH-specific fine motor milestones<br>Achievement of ACH-specific gross motor milestones<br>Achievement of ACH-specific communication milestones<br>Pain control<br>Independence in self-care, including toileting<br>Functionality<br>Improvement in health-related quality of life factors<br>Proportionality<br>Increased height<br>Ability to take part in physical activity<br>Improvement in gait<br>Resolution of spinal thoracolumbar kyphosis once mobile<br>Resolution of complications associated with sleep-disordered breathing<br>Resolution of otitis media<br>Resolution of middle ear effusion<br>Psychosocial – acceptance and understanding of their condition		

Educational – supporting provision of appropriate support in school (physical, psychological, educational)		

Please provide comment on your response:
