# Supplementary material for: Recommendations for management of infants and young children with achondroplasia: Does clinical practice align?
Source: Orphanet J Rare Dis. 2025 Mar 11;20:114. doi: 10.1186/s13023-025-03621-7 (PMC11895228; doi:10.1186/s13023-025-03621-7)
Supplement: Supplementary file 2 — Additional file 2: Clinical management challenges in infants and young children with achondroplasia—Parent survey. [file 13023_2025_3621_MOESM2_ESM.pdf]

EAF
European Achondroplasia Forum

Clinical management challenges in infants and young children with achondroplasia – Parents

Survey Objectives

- Identify the key challenges faced by families in the care provided by healthcare professionals for their infants/young children
- Identify the key outcomes of importance for families of children with achondroplasia
- Assess the alignment between care received and recommendations

Please answer questions based on your own personal experience. If you are aware of people with different experiences, please describe these in the Comments. If you have more than one child with achondroplasia, please repeat the survey for each child.

General

1. Please enter your country**:**

2. Are you:
- ☐ An individual of average stature
- ☐ An individual with achondroplasia
- ☐ An individual of average stature with a partner with achondroplasia

Comment:

3. How old is your child?
- ☐ ≤5 years of age
- ☐ 5–11 years
- ☐ 12–17 years
- ☐ 18+ years

Comment:

4. In what healthcare setting is your child predominantly managed?
- ☐ Academic institution/Specialist achondroplasia centre/University hospital
- ☐ General hospital
- ☐ Primary care
- ☐ Other, please specify

Key challenges in infants and young children

5. Thinking about the care provided by healthcare professionals, which three complications of achondroplasia experienced by your child as an infant (aged ≤2 years) have you/your family found the most challenging?

	Tick THREE options
Sleep-disordered breathing	
Otitis media	
Middle ear effusion	
Foramen magnum stenosis	
Thoracolumbar kyphosis	
Spinal stenosis	
Kyphosis	
Genu varum	
Obesity/weight management	
Health-related quality of life factors (e.g., independent living, adaptive measures)	
Other (please specify):	

Please provide comment on why these complications have been challenging:

6. Thinking about the care provided by healthcare professionals, which three complications of achondroplasia experienced by your child in early childhood (aged 2–5 years) have you/your family found the most challenging?

	Tick THREE options
Sleep-disordered breathing	
Otitis media	
Middle ear effusion	
Foramen magnum stenosis	
Thoracolumbar kyphosis	
Spinal stenosis	
Kyphosis	
Genu varum	
Obesity/Weight management	
Health-related quality of life factors (e.g., independent living, adaptive measures)	
Other (please specify):	

Please provide comment on why these complications have been challenging:

7. After treatment/therapies/surgeries for the following complications, what were the results?

	Completely resolved	Somewhat resolved	No change	Slightly worsened	Significantly worsened
Sleep-disordered breathing<br>Otitis media<br>Middle ear effusion<br>Foramen magnum stenosis<br>Thoracolumbar kyphosis<br>Spinal stenosis<br>Kyphosis<br>Genu varum<br>Obesity/weight management<br>Health-related quality of life factors (e.g., independent living, adaptive measures)<br>Other (please specify):					

Please provide comment on your response:

Recommendations and clinical practice

8. Prior to taking this survey, how aware were you of the existence of the EAF Guiding Principles for Management of Achondroplasia and the International Consensus Recommendations for the Management of Achondroplasia?

	EAF Guiding Principles	International Consensus Recommendations
Aware it existed, and have read it<br>Aware it existed but have not read it<br>Not at all aware		

Please comment:

Outcomes in infants and young children

9. From your perspective as a parent of a child with achondroplasia, what do you consider to be the most important outcomes in the management of achondroplasia in infants (aged ≤2 years)? Please select up to three options.

	Tick THREE options
Decompression of the foramen magnum (if indicated)	
Achievement of ACH-specific growth milestones	
Achievement of ACH-specific fine motor milestones	
Achievement of ACH-specific gross motor milestones	
Achievement of ACH-specific communication milestones	
Pain control	
Independence in self-care, including toileting	
Functionality	
Improvement in health-related quality of life factors	
Proportionality	
Increased height	
Ability to take part in physical activity	
Improvement in gait	
Resolution of spinal thoracolumbar kyphosis once mobile	
Resolution of complications associated with sleep-disordered breathing	
Resolution of otitis media	
Resolution of middle ear effusion	
Psychosocial – acceptance and understanding of their condition	
Educational – supporting provision of appropriate support in school (physical, psychological, educational)	

Please provide comment on your response:

10. From your perspective as a parent of a child with achondroplasia, what do you consider to be the most important outcomes in the management of achondroplasia in young children (aged 2–5 years)? Please select up to three options.

	Tick THREE options
Decompression of the foramen magnum (if indicated)	
Achievement of ACH-specific growth milestones	
Achievement of ACH-specific fine motor milestones	
Achievement of ACH-specific gross motor milestones	
Achievement of ACH-specific communication milestones	
Pain control	
Independence in self-care, including toileting	
Functionality	
Improvement in health-related quality of life factors	
Proportionality	
Increased height	
Ability to take part in physical activity	
Improvement in gait	
Resolution of spinal thoracolumbar kyphosis once mobile	
Resolution of complications associated with sleep-disordered breathing	
Resolution of otitis media	
Resolution of middle ear effusion	
Psychosocial – acceptance and understanding of their condition	
Educational – supporting provision of appropriate support in school (physical, psychological, educational)	

Please provide comment on your response:
